# metaGEENOME: an integrated framework for differential abundance analysis of microbiome data in cross-sectional and longitudinal studies

**DOI:** 10.1186/s12859-025-06217-x

**Published:** 2025-07-21

**Authors:** Ahmed Abdelkader, Nur A. Ferdous, Mohamed El-Hadidi, Tomasz Burzykowski, Mohamed Mysara

**Affiliations:** 1 Bioinformatics Research Group, Center for Informatics Science (CIS), School of Information Technology and Computer Science (ITCS), Nile University, Giza, Egypt; 2 Department of Cancer and Genomic Sciences, School of Medical Sciences, College of Medicine and Health, University of Birmingham Dubai, Dubai, United Arab Emirates; 3 Data Science Institute, Hasselt University, Hasselt, Belgium; 4 Department of Biostatistics and Medical Informatics, Medical University of Bialystok, Bialystok, Poland; 5 International Drug Development Institute (IDDI), Louvain-la-Neuve, Belgium

**Keywords:** Microbiome, Differential abundance, Biomarkers, Repeated measures, False discovery rate

## Abstract

**Background:**

Detecting biomarkers is a key objective in microbiome research, often done through 16S rRNA amplicon sequencing or shotgun metagenomic analysis. A critical step in this process is differential abundance (DA) analysis, which aims to pinpoint taxa whose abundance significantly differs between groups. However, DA analysis remains challenging due to high dimensionality, compositionality, sparsity, inter-taxa correlations, uneven abundance distributions, and missing values—all which hinder our ability to model the data accurately. Despite the availability of many DA tools, balancing high statistical power with effective false discovery rate (FDR) control remains a major limitation.

**Results:**

Here, we introduce a novel approach for DA analysis that integrates counts adjusted with Trimmed Mean of M-values (CTF) normalization and Centered Log Ratio (CLR) transformation with Generalized Estimating Equation (GEE) model. We benchmarked our approach against eight widely used tools employing both simulated and real datasets in cross-sectional and longitudinal settings. While several tools (e.g. MetagenomeSeq, edgeR, DESeq2 and Lefse) achieved high sensitivity, they often failed to adequately control the FDR. In contrast, our method demonstrated high sensitivity and specificity when compared to other approaches that successfully controlled the FDR, including ALDEx2, limma-voom, ANCOM, and ANCOM-BC2.

**Conclusions:**

Our approach effectively addresses key challenges in microbiome data analysis across both cross-sectional and longitudinal designs. Integrated into the R package metaGEENOME (https://github.com/M-Mysara/metaGEENOME), our framework provides a flexible, scalable and statistically robust solution for DA analysis, offering improved FDR control and enhanced performance for biomarker discovery in microbiome studies.

**Graphical abstract:**

**Supplementary Information:**

The online version contains supplementary material available at 10.1186/s12859-025-06217-x.

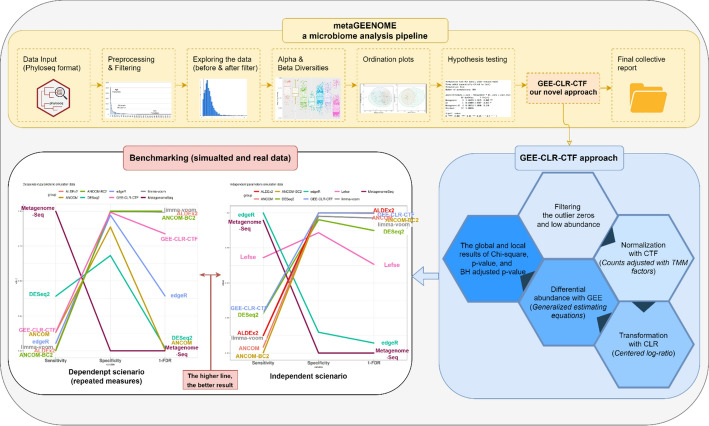

## Background

Within the field of microbiome research, understanding the differential abundance (DA) of bacterial species provides essential insights into their ecological and functional roles, shedding light on the complex interactions between microorganisms and their environments [1]. High-throughput sequencing technologies, including 16S ribosomal RNA gene sequencing (targeting hypervariable regions such as V1–V3 or V4–V5) and shotgun metagenomics, have become the foundation of microbial community profiling [2]. There is a growing demand for analytical approaches capable of identifying differentially abundant microbes as potential biomarkers for various applications, including early disease diagnosis [3]. Such methods help unravel the intricate network of microbial interactions and their consequences for broader biological systems [4].

Microbial sequencing primarily yields relative abundance data, which reflects the proportion of individual species rather than the absolute magnitude of the microbial population. This inherent compositionality introduces analytical challenges: a change in the absolute abundance of one species consequently affects the read-out of the other species within the same sample, potentially leading to misinterpretation of differential abundance [5]. Additionally, microbial datasets often exhibit high sparsity, with overinflation of zeros. These zeros may arise from biological absence (structural zeros), low sequencing depth (sampling zeros), or outliers [6]. Further complexity arises due to intra-/inter-taxa correlations, especially in studies with repeated measurements or longitudinal designs. Challenges such as high dimensionality (more taxa than samples), unbalanced coverage (differences in the sequencing depth), and imbalance between bacterial species (as such exists naturally in low- high abundances) vary across studies and can significantly influence analytical outcomes [7]. Effectively addressing these complexities is crucial to properly identify bacterial biomarkers.

To confront these issues, numerous approaches have been developed. Initially, methodologies originally designed for RNASeq data has been suggested, including the DESeq2 and edgeR [8, 9] strategies leveraging the negative binomial distribution. The strategies incorporate, respectively, the modified Relative Log Expression (RLE) and Trimmed Mean of M-values (TMM) normalization without any transformations. Alternatively, dedicated approaches for microbial data analysis have been proposed. For instance, MetagenomeSeq [10] adopts zero-inflated normalization, and supplements it with Cumulative Sum Scaling (CSS) normalization together with the logarithmic transformation. Lefse [2] applied a non-parametric Kruskal–Wallis (KW) sum-rank test and the pairwise Wilcoxon rank-sum test, and uses linear discriminant analysis (LDA) to estimate the effect sizes. ALDEx2 [11] relies exclusively on the centered log-ratio (CLR) transformation, ANCOM applies the additive log-ratio (ALR) transformation, while limma-voom combines TMM with the logarithmic transformation [12]. Despite these efforts, benchmarking studies (e.g., Hawinkel et al*.* [13]) reveal that no single tool achieves an optimal balance between statistical power to detect DA and effective control of the false discovery rate (FDR). This highlights a persistent gap in the field and underscores the need for innovative approaches that can improve the trade-off between sensitivity, specificity, and FDR control in DA analysis.

Generalized Estimating Equations (GEE) is a statistical method widely applied in various biological analyses, including correlated microbial sequencing studies [14], quantification of polygenic effects [15], analysis of time-course gene sets [16], detection of sparse microbial association signals [17], and modifications of variance estimators [18]. GEE is particularly suitable for longitudinal or clustered microbiome data, as it accounts for within-subject correlations and supports distribution-flexible modeling, facilitating robust identification of differentially abundant taxa in both cross-sectional and longitudinal settings. In this study, we propose a GEE-based framework, integrated with suitable normalization and transformation strategies, for microbiome data analysis. We conduct a benchmarking analysis comparing our approach to other existing methods in microbial biomarker detection. For this purpose, we use both simulated and real datasets capturing both cross-sectional and longitudinal designs. We consolidate our downstream analysis steps for microbial data into a unified R package named metaGEENOME, with the objective of improving the analytical framework in this field.

## Methods

### GEE-CLR-CTF model

We aim to develop an approach capable of addressing the challenges associated with microbial differential expression analysis. The approach consists of data normalization, transformation, and modelling. Regarding the normalization, a comparative analysis was performed using microbial data between various normalization techniques such as CPM (counts per million), QNT (quantile), UQ (upper quartile), and CTF (Counts adjusted with Trimmed Mean of M-values) [19]. The CTF normalization that assumes the majority of taxa display no differential abundance (DA) between the samples accounting for library size variability has demonstrated the highest performance (data not shown). Computationally, CTF involves obtaining library size-normalized read counts for each taxon in every sample, and calculating the log2 fold change (M value) between two samples as follows:

1 $$M={log}_{2}\frac{treated sample count}{control sample count}$$

Simultaneously, the absolute expression count (A value) is derived as follows:

2 $$A=\frac{{log}_{2}\left(treated sample count\right)+{log}_{2}(control sample count)}{2}$$

Further processing entails double-trimming the upper and lower percentages of the data (trimming M values by 30% and A values by 5%). The weighted mean of M values is obtained after trimming, leading to the calculation of the normalization factor [20].

Regarding the transformation which is a vital part of our approach, we note that microbial data exist in the Aitchison simplex sample space (S); hence, we focus on meaningful ratios between components (relative abundance) rather than the absolute abundance [21]. Through log-ratio transformations, it is possible to transition this compositional data from S to a conventional multivariate problem in the real vector space ${R}^{D-1}$ [22, 23]. For this purpose, we have considered two suitable transformation methods namely additive log-ratio (ALR) and centered log-ratio (CLR). The formal is subject to a consistency issue as it requires the identification of a reference taxon, which should remain unchanged in absolute counts across samples [24]. However, detecting such a reference taxon in relative abundance data is challenging and often unreliable, as it typically involves arbitrary selection or ranking methods [5]. In contrast, CLR transformation avoids the need for a reference taxon by averaging the taxa, thereby providing more robust results. The superiority of CLR was further confirmed when conducting the comparative analysis between both approaches. While both approaches achieved similar levels of specificity, controlling the FDR was only possible with the CLR approach, which was also capable of achieving double the sensitivity level achieved with ALR transformation (Supplementary Material 1). The CLR equation below represents the ratio of abundance of each taxon ${x}_{n}$ divided by the geometric mean of all taxa in every sample $G\left(x\right)$:

3 $$CLR\left(x\right) = \left\{log \left(\frac{{x}_{1}}{G\left(x\right)}\right),\dots,log \left(\frac{{x}_{n}}{G\left(x\right)}\right) \right\}= \left\{log \left({x}_{1}\right) -log \left(G\left(x\right)\right),\dots,log \left({x}_{n}\right) -log \left(G\left(x\right)\right) \right\}$$

As for the modelling, we employed the Generalized Estimation Equation (GEE) method, introduced by Liang and Zeger in 1986 [25], to estimate without fully specifying the likelihood function, making it ideal for handling complex multivariate categorical responses [26]. GEE remains robust in estimating regression parameters even when the correlation structure is misspecified and offers flexibility in modeling mean structures across different regression techniques. It can also handle missing data, provided that the data are missing completely at random (MCAR) [25, 27–31]. As an alternative, one could consider the use of the generalized linear mixed models (GLMMs) [32]. However, applying GLMMs for distributions other than the normal one is computationally challenging because it requires the use of numerical integration to compute the likelihood function. Also, GLMMs results lead to subject-specific interpretation, while GEE models can be interpreted in terms of population averages. Hence, we consider GEE as the better choice for our count-based abundance data, enabling efficient parameter estimation without the complexities associated with GLMM [33].

When defining the GEE models, we opted for a compound symmetry (exchangeable) working correlation structure which assumes equal correlation across all measurements from each subject. Admittedly, other structures could be considered (e.g., independent, unstructured, or autoregressive). However, GEE provides consistent parameter estimates even if the working correlation structure is misspecified [34]. The compound symmetry structure offers a reasonable trade-off between the numerical complexity and the efficiency of the estimation. This was further confirmed by a sensitivity analysis that compared the results obtained for the structure with those yielded by the GEE with the independence, unstructured, and autoregressive working correlations (data not shown).

We consider a repeated measures setting, in which $K$ taxa are measured for $N$ individuals at $J$ different occasions, with individuals grouped in $G$ groups. Note that this setting also includes the simplest case of an experiment with a single measurement. The general GEE formulation can be written as the following estimating equation for coefficients $\beta$ of a particular model:

4 $$U\left(\beta \right)= \sum_{i=1}^{N}{\left(\frac{\partial {\mu }_{i}}{\partial \beta }\right)}^{T}{V}_{i}^{-1}\left({Y}_{i}-{\mu }_{i}\right)=0$$

In Eq. 4, $U(\beta )$ is the estimating equation for the parameter vector $\beta$, ${Y}_{i}$ represents the vector of observed responses for the $i$-th subject, ${\mu }_{i}={g}^{-1}({X}_{i}\beta )$ is the expected mean vector where ${g}^{-1}$ is the inverse of the link function [33], and ${X}_{i}$ is the matrix containing the values of the explanatory variables for the $i$-th subject, and ${V}_{i}$ is the working variance–covariance matrix for the $i$-th subject (derived from the chosen working correlation structure).

We apply the GEE approach to the following model:

5 $$E\left({Y}_{ijg}^{k}\right)={\mu }_{ijg}^{k}={\beta }_{0}^{k}+{\beta }_{1,g}^{k}+{\beta }_{2,j}^{k}+{\beta }_{3,jg}^{k}$$

where ${Y}_{ijg}^{k}$ denotes the CLR- transformed count of the ${k}^{th}$ taxon for the ${i}^{th}$ individual from the ${g}^{th}$ group measured at the ${j}^{th}$ occasion. The interest lies in the regression coefficients $\beta$. We assume that ${\beta }_{\text{1,1}}^{k}={\beta }_{\text{2,1}}^{k}={\beta }_{\text{3,11}}^{k}=\dots ={\beta }_{3,G}^{k}={\beta }_{\text{3,21}}^{k}=\dots ={\beta }_{\text{3,2}G}^{k}=0$, so that ${\beta }_{0}^{k}$ captures the mean value of the CLR- transformed count of the ${k}^{th}$ taxon for the first (reference) group at the first (baseline) occasion, ${\beta }_{1,\text{g}}^{k}$ captures the change of the mean value for the ${g}^{th}$ group (i.e., the group effect), ${\beta }_{2,\text{j}}^{k}$ captures the change of the mean value at the ${j}^{th}$ occasion (i.e., the time effect), and ${\beta }_{3,\text{jg}}^{k}$ captures the additional (interaction) change specific for the ${g}^{th}$ group at the ${j}^{th}$ occasion (i.e., the group x time interaction effect). Furthermore, it is assumed that ${Y}_{ijg}^{k}$ follows a normal distribution with mean $E\left({Y}_{ijg}^{k}\right)$ and that all $KJ$ measurements (for all taxa and occasions) for the ${i}^{th}$ individual are equicorrelated; in other words, a compound-symmetry (exchangeable) working correlation structure is assumed. Note that Eq. 5 implies the use of an identity link function, i.e., $g\left({\mu }_{ijg}^{k}\right)={\mu }_{ijg}^{k}$.

### Implementation of the GEE-CLR-CTF approach

**Fig. 1:**
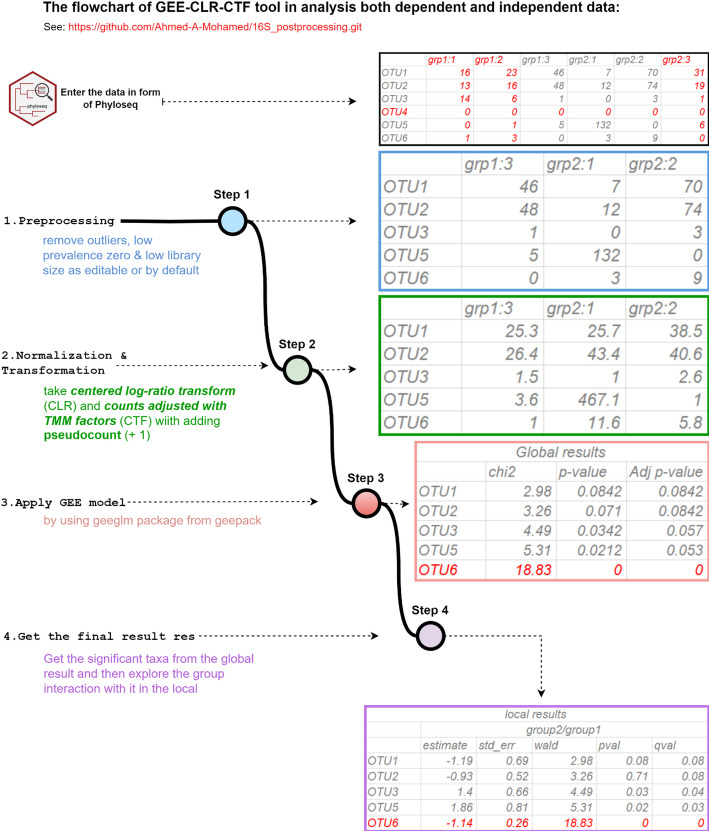
Illustration of workflow of GEE-CLR-CTF for microbial differential abundance (DA) analysis. The flow chart outlines the steps for analyzing microbiome DA under both cross-sectional and longitudinal data scenarios using GEE-CLR-CTF that as an example we have 3 samples in group 1 (grp1:1, grp1:2, grp1:3) and 3 samples in group 2 (grp2:1, grp2:2, grp2:3). Utilizing the *Phyloseq* R package (V 1.40.0), the workflow involves: (1) Preprocessing step, including removing outliers, low-prevalence taxa, samples with low library size, and accounting for zero inflation, such as taxa and samples marked with red above. (2) Regarding for the compositional nature of data, the filtered data is transformed using the combination of CTF with CLR methods after adding 1 to the abundance as pseudocount to prevent the error of logarithmtic zero. (3) The GEE model is then applied to the transformed data, accomplished using *geepack* R package (V 1.3.9). 4) The result of the GEE model consists of local coefficients for taxa and their interactions with various variables. These local results are then used to create a global result. Both the global and local tables provide statistical tests such as Wald or chi-squared tests, along with *p* value

Our primary objective is the development of a novel approach designed to identify bacterial biomarkers by addressing the challenges associated with microbial differential expression analysis. These challenges involve several key stages: preprocessing, normalization, transformation, GEE model, and putting it all together in the final output. Preprocessing is aimed at eliminating outliers, low-abundance entries, and outlier zeros while retaining both sampling and structural zeros for later processing. This is accomplished by evaluating the distribution of transformed count data within each group and differentiating between standard data points and possible outliers through following preprocessing steps adapted from ANCOM-II [6]. Normalization with CTF is followed by adding a pseudo-count to solve the problem of the logarithmic transformation of zeros, and transformation with CLR. This should bring the data closer to normality and tackle the between-sample error and the compositionality issues. Subsequently, the GEE Model is employed by using the *geepack* R package, enabling robust statistical DA analysis for datasets of both independent and dependent (repeated measures) experimental set-ups. This model can comprehend diverse distributions and correlation patterns while effectively handling missing values. The Final output includes both global and local results, providing vital statistical metrics like P-values and Wald tests. The global results indicate the taxa that are significant overall data, while the local results enquire into the interaction of these taxa with the variable groups. These steps were combined in a single function entitled GEE-CLR-CTF within our developed *metaGEENOME* package in R. A visual representation of GEE-CLR-CTF function illustrated in Fig. 1, providing a comprehensive understanding of the DA patterns and their associations within the dataset.

### Data sources

For benchmarking purposes, we used both simulated and real-life data. Regarding the cross-sectional setting, we considered the Human Microbiome Project (HMP), which uses Roche-454 FLX Titanium sequencing for the 16S rRNA gene’s V1–V3, V3–V5, and V6–V9 variable regions. Data preprocessing involved QIIME software encompassing steps like retrieving SFF, mapping files from the project's website, and cleaning via PrimerProspector. Subsequent steps included operational taxonomic unit (OTU) selection, chimera checking, clustering OTUs at a 97% similarity threshold, and taxonomic assignment. We utilized 464 human samples from various body sites, including stool, tongue dorsum, and mid-vagina and included only the health group [35]. Additionally, we considered the American Gut Project (AGP), which provided a database of human gut microbiome featuring 16S rRNA V4 gene fragments from Illumina sequencing. The sequences were processed by Deblur v1.0.2, involving trimming and integrating into the Greengenes reference tree. Taxonomic assignment was performed by using the RDP classifier within QIIME2. Therefore, we used 2621 human fecal samples from both health and patients with inflammatory bowel disease (IBD) [36].

For the repeated measures setting, we considered the Pelvic Irradiation study which investigated microbiome responses and intestinal mucositis induced by pelvic radiotherapy. The samples were sequenced for the V3–V4 hypervariable region using the Illumina platform. We used 20 mice fecal samples from non-radiated control group (Saline_0) within time points week 0 and week 3 [37]. The ANTICIPATE study examined intestinal microbiota in Clostridioides difficile hospitalized patients. The samples from patients were subjected to 16S rRNA sequencing gene through the Illumina protocols, primarily targeting the V3–V5 regions. We used 654 human fecal samples from two infectious groups (J01C and J01D) within time points day 1 and day 6 [38]. Both studies employed the OCToPUS v1.0 pipeline for preprocessing, involving de-noising, contig creation, alignment of retained contigs with the SILVA database, elimination of contigs outside the V3–V4 region with high homopolymers using the Illumina Paired-End Denoiser algorithm, de novo chimera removal, and OTU clustering, followed by classification by the Ribosomal Database Project (RDP).

### Simulated and real-life data setup

Comprehensive benchmarking assessments require the utilization of both simulated and real data, each addressing the limitations of the other. Simulated data offer an optimal approach for sensitivity detection due to the knowledge of the ground truth. Benchmarking using real-life data is a standard practice for tools without artificial processes, despite the lack of precision in its ground truth evaluation that might result in an inflated number of false positives and false negatives.

Simulated data were obtained by introducing a threefold changes in abundance for selected taxa in the health group using the negative binomial distribution in both up and down-regulation. Subsequently, multiple sub-data were created by randomly selected taxa and shuffling between the group of the simulated data. Additionally, to ensure the robustness of simulated data results, efforts were made to counter naivety and ensure data cleanliness by introducing outliers and establishing correlations between taxa. Real-life data were created through sub-dataset division and comparison against verified truth. The additional details are provided in the Supplementary Material 4.

To ensure data reliability, we excluded low-prevalent taxa present in less than 10% of the samples. Following the logic outlined by the analysis of Hawinkel et al. [13], we adjusted our benchmarking approach to account for both the repeated measures and cross-sectional scenarios. In cross-sectional settings, we employed HMP in simulated data to generate 1000 sub-datasets, with each including 100 randomly selected taxa, 50 shuffled samples, and two groups (healthy and simulated). For real data, the AGP was used, resulting in 750 sub-datasets, consisting of 100 randomly selected taxa, 250 shuffled samples, and two groups (healthy and IBD). Each sub-dataset was split into three smaller sets to evaluate the results of significant taxa within the smaller set by comparing them to the verified truth taxa of the entire sub-dataset.

Additionally, for repeated-measures settings, we retained the same assumptions as before, but with modifications to fit into the longitudinal data. The Pelvic Irradiation data was employed in simulated data, derived from mice dataset, aimed at reducing the complexity of confounders during data simulation. The Saline_0 group was selected due to its stable abundance across time points, generating 100 sub-datasets with 100 random taxa, 40 shuffled samples, and complex metadata, including two groups (Saline_0 and simulated) across two-time points (week 0 and week 3). Also, ANTICIPATE study was used in real data, featuring two groups (J01C and J01D) and two-time points (day 1 and day 6) divided into 58 verified sub-data with 300 shuffled samples and 100 random taxa, divided into three parts evaluated data, as previously described.

To address various challenges in microbial data analysis, we simulated cross-sectional HMP data, using the same method described earlier, by adjusting the parameters to simulate these specific challenges. Outliers and sampling artifacts were controlled by adjusting Pearson residuals (up to values of 3, 4, or 5) based on library sizes, with lower values leading to more induced outliers. To introduce greater complexity in the data dimensions, we expanded the number of taxa in the dataset to include 100, 150, and 200 taxa. Unbalanced sequencing coverage was modeled by varying the sequencing depth, ranging from low coverage (half the depth) to high coverage (double the depth). To simulate abundance imbalances, taxa were classified into three groups: low abundance (< 10%), intermediate abundance (10%-90%), and high abundance (> 90%). Zero inflation and sparsity were examined by manipulating the effect size (fold changes of 2, 3, and 4), and by comparing data scenarios with and without compensation (upregulation versus both upregulation and downregulation). For datasets incorporating compensation, structural zeros were introduced by reducing actual abundance values to log-negative values. The default parameters for each sub-dataset simulation included 50 samples, 100 taxa, Pearson residuals below 5, a threefold change in effect size, and zero-inflated compensatory data.

### Benchmarking design and tool comparison

The study aimed to evaluate the GEE-CLR-CTF model's performance against established DA tools in both cross-sectional and repeated-measures settings through the application to simulated and real-life data. We included in our evaluation tools that assume binomial distribution such as edgeR (V 3.40.0) [20], DESeq2 (V 1.38.0) [8], and MetagenomeSeq (V 1.40.0) [10]. Moreover, we also included tools based on the Poisson distribution including limma (V 3.54.0) [39]. Additionally, ALDEx2 (V 1.30.0) [11], ANCOM (ANCOMBC V 2.0.1) [40], and ANCOMBC2 (ANCOMBC V 2.0.1), Lefse (lefser V 1.14.0) [2] were considered (Table 1).

**Table 1:** Comparing microbial differential abundance tools used in the benchmarking

Tool (version)	Distribution	Statistical model	Normalization	Transformation	Repeated measures
GEE-CLR-CTF (0.1.0)	Poisson	GEE	CTF	CLR	Yes
ANCOM (2.0.1)	Non-parametric	ANOVA, Mixed-effects model	None	ALR	Yes
ALDEx2 (1.30.0)	Dirichlet-multinomial	ANOVA, t-test	None	CLR	No
limma-voom (3.54.0)	Normal	linear modelling	log-CPM	log	Yes
MetagenomeSeq (1.40.0)	zero-inflated normalization	zero-inflated mixture model	CSS	log	No
edgeR (3.40.0)	Negative binomial	exact test	TMM	None	No
DESeq2 (1.38.0)	Negative binomial	generalized linear model	RLE	None	No
Lefse (lefser 1.14.0)	Non-parametric	Kruskal–Wallis	CPM	None	No

Performance criteria included sensitivity TP/(TP + FN), specificity TN/(TN + FP), and false discovery rate (FDR) FP/(FP + TP), computed based on true positive (TP), true negative (TN), false positive (FP), and false negative (FN) counts. We sought a tool characterized by high sensitivity, high specificity, and low FDR. Performance results were analyzed by using the Kruskal–Wallis test followed by post hoc pairwise Wilcoxon rank-sum tests to identify significant differences. The Benjamini-Hochberg (BH) procedure was applied to adjust *p* values for multiple testing [41]. The adjusted two-sided *p* values were evaluated by using the 0.05 level.

## Results

### Cross-sectional setting

**Fig. 2:**
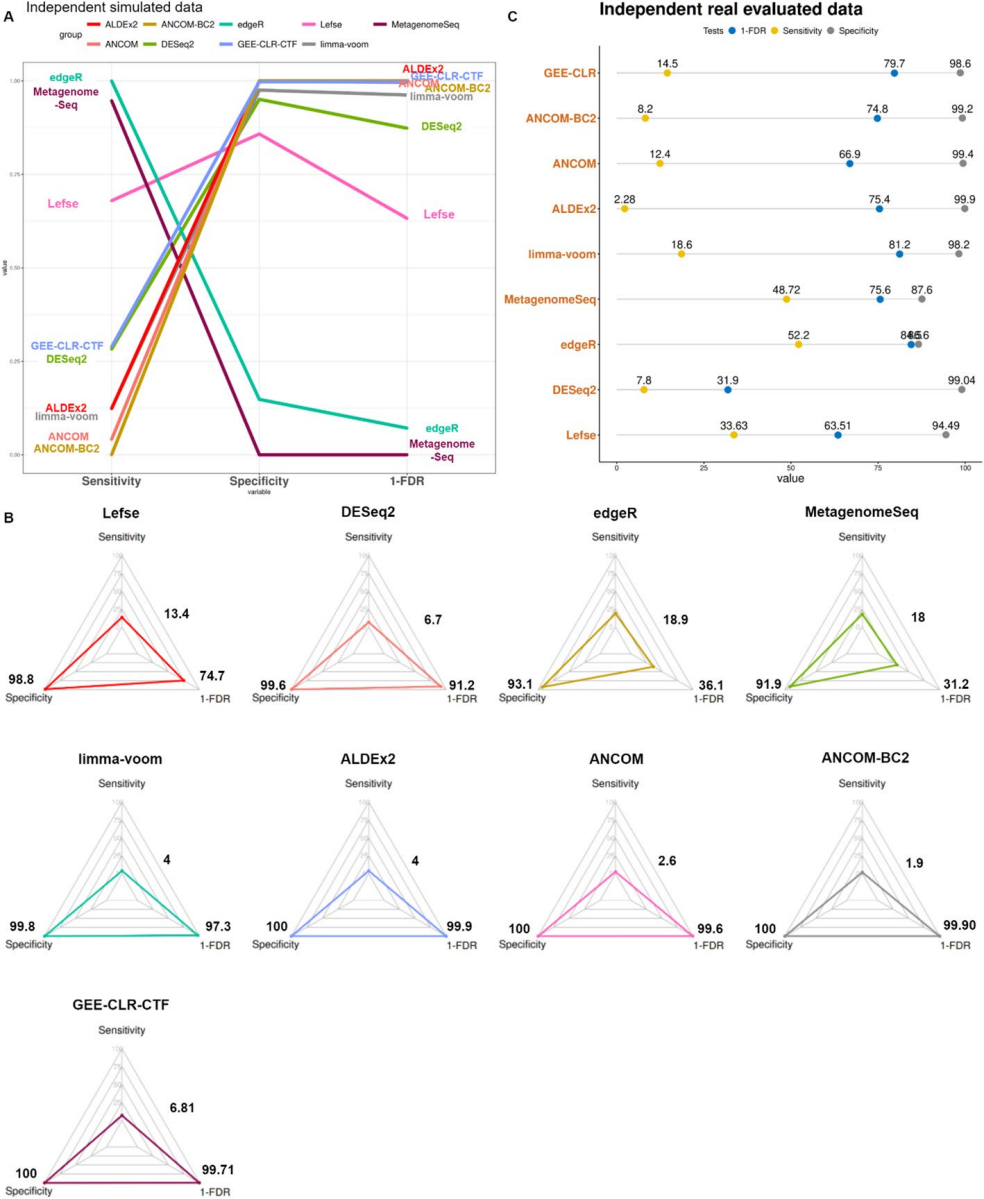
Evaluation of differential abundance tools in cross-sectional simulated and real evaluated data. Panels illustrate comparisons in sensitivity, specificity, and 1-FDR (the reverse of FDR values) across tools for cross-sectional data analysis, tested in both simulated and real scenarios. **A** The parallel coordinate plot demonstrates tool performance, where higher levels of sensitivity, specificity, and 1-FDR indicate better performance. Lefse, MetagenomeSeq and edgeR display high sensitivity but lower specificity and fail to control 1-FDR (< 95%), while DESeq2 follows with moderate sensitivity and high specificity without controlling 1-FDR. GEE-CLR achieves balanced performance with comparatively high sensitivity and excellent control over specificity and 1-FDR. Despite the other tools exhibiting good controlling over FDR and specificity, they have very low sensitivity level **B** The radar chart reinforces these findings, illustrating improved performance with larger triangle areas. **C** Real data assessment reveals tool performance in sensitivity, specificity, and 1-FDR panels, independent of ground truth knowledge. Lefse, MetagenomeSeq and edgeR demonstrate high sensitivity but lower specificity, while limma-voom, ANCOM with a detection rate of 0.6, and GEE-CLR-CTF exhibit well-balanced performance across these parameters

For the simulated data, the results reflect the average across 1000 sub-datasets created from the HMP data. Each sub-dataset included 100 taxa, 50 samples, and two groups. Subsequently, the results of all methods were compared using the Kruskal–Wallis test followed by post hoc pairwise Wilcoxon Rank Sum tests. Lefse, MetagenomeSeq, and edgeR demonstrated sensitivity averaging at 16.7% and specificity averaging at 94.6%, while failing to control the FDR (averaging at 52.7%). DESeq2 achieved a sensitivity of about 6.7% and a specificity of 99.6% without proper control of FDR (8.8%). The other methods such as GEE-CLR-CTF, ANCOM, ANCOM-BC2, ALDEx2, and limma-voom displayed exceptional specificity levels, averaging above 99.9%, while maintaining the FDR levels at an average of 0.7%, and achieving a sensitivity of about 3.9% on average. Interestingly, the GEE-CLR-CTF model exhibited a mean sensitivity level of 6.8% which was significantly higher when compared to other methods able to control the FDR (BH adjusted *p* value from ${5.3\times 10}^{-16}$ into 0.00021 in pairwise Wilcoxon Rank Sum tests) while conservatively controlling the FDR at 0.3% (adjusted p-value from ${2\times 10}^{-16}$ into 0.0074 in Wilcoxon Rank) and yielding a specificity of 99.7% (Fig. 2A, Supplementary Material 2).

For the real-life data, the ground truth was inferred, as proposed by Hawinkel et al. [13], based on coherence between the findings obtained by using the complete data and a subset of the data. The 750 examined sub-datasets, derived from AGP, consisted of 100 taxa, 250 samples, and two groups. As expected with the tools not able to control the FDR because of assuming amount of significant taxa, Lefse, MetagenomeSeq, and edgeR achieved a sensitivity level averaging 44.9% and specificity level averaging 89.2%. The remaining methods yielded substantially lower sensitivity around, on average, 10%, while offering a specificity of about 100% on average. GEE-CLR-CTF yielded a sensitivity of 14.5% and specificity of 98.6%, close to ANCOM and limma (Supplementary Material 2) and similar to its performance in the simulated data. Interestingly, most tools managed to adequately control the FDR, except for DESeq2 (Fig. 2B).

### Repeated-measures setting

**Fig. 3:**
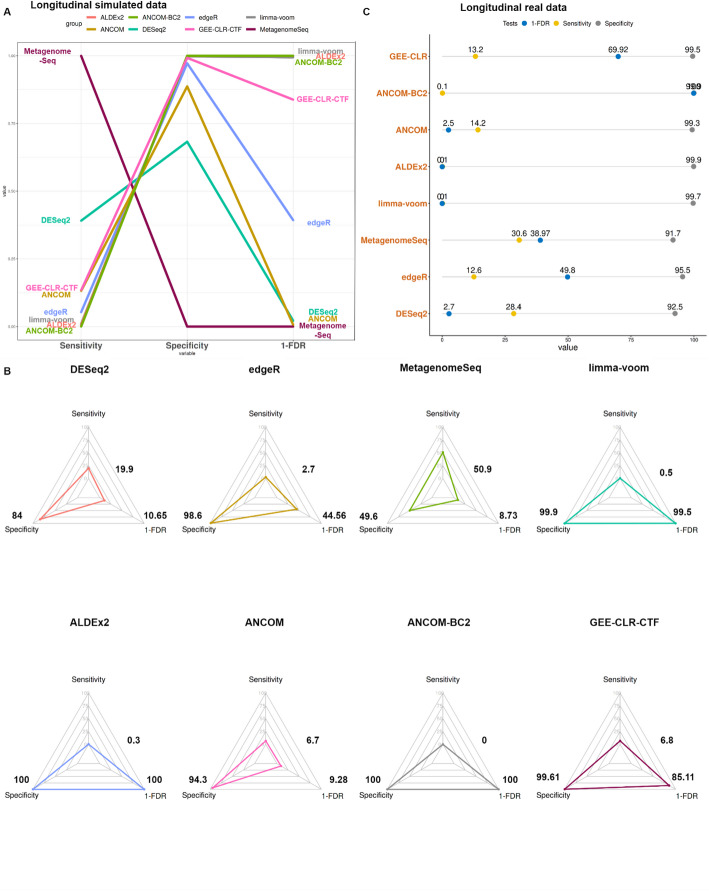
Evaluation of differential abundance tools in longitudinal simulated and real evaluated data. The figures present comparative analyses of sensitivity, specificity, and 1-FDR (the inverse of FDR values) across various tools for dependent data assessment, evaluated in both simulated and real set-ups. **A** In simulated data, MetagenomeSeq and DESeq2 have high sensitivity level without controlling either specificity or FDR. While many tools, namely limma-voom, ALDEx2, and ANCOM-BC2, show no sensitivity, others demonstrate higher sensitivity and specificity with low level FDR. GEE-CLR-CTF has balance in achieving high sensitivity, high specificity and low level FDR. **B** The radar chart reinforces these findings, illustrating improved performance with larger triangle areas. **C** In real-evaluated data, many tools fail to control FDR, such as DESeq2, limma-voom, ALDEx2, and ANCOM, while ANCOM-BC2 has no sensitivity level. GEE-CLR-CTF outperforms MetagenomeSeq and edgeR due to its ability to maintain high levels of specificity and 1-FDR with achieving a good level of sensitivity

For the simulated data, we employed the Pelvic Irradiation study to create 100 sub-datasets with 100 taxa, 40 samples, two groups, and two-time points. MetagenomeSeq and DESeq2 demonstrated sensitivity levels of about 50.9% and 19.9%, respectively. Their specificity was equal to about 49.6% and 84%, respectively. They exhibited poor control of the FDR (averaged 90.3%). The remaining methods achieved specificity above 95%, but not all of them, except of GEE-CLR-CTF, could achieve a balance between sensitivity and the control of the FDR. For instance, edgeR yielded a sensitivity of 2.7% with the FDR of 55.4%, while ANCOM gave a sensitivity of 6.7% and the FDR of 90.7%. For limma-voom, ALDEx2, and ANCOM-BC2, sensitivity was below 1%, but the FDR was adequately controlled. The GEE-CLR-CTF achieved a sensitivity of 6.8% (adjusted p-value from 0.000000088 into 0.0000073 in Wilcoxon Rank), specificity 99.6% (adjusted *p* value from ${2\times 10}^{-16}$ into 0.000008 in Wilcoxon Rank), and the FDR of 14.9% (adjusted *p* value from 0.0000041 into 0.000017 in Wilcoxon Rank) (Fig. 3A, Supplementary Material 2).

For the real-life data, ANTICIPATE dataset was utilized to generate 58 sub-datasets with 100 taxa, 300 samples, two groups, and two time points. MetagenomeSeq and DESeq2 exhibited sensitivity levels similar to those observed in the simulated data (about 29.5%), with an average specificity of 92.1%. GEE-CLR-CTF, EdgeR, and ANCOM yielded lower sensitivity, averaging 13.3%. The sensitivity of the remaining methods was even lower (at most 0.1%). All the methods approached almost 100% specificity. The FDR was the lowest for GEE-CLR-CTF (30%, adjusted *p* value from ${2\times 10}^{-16}$ into 0.0175 in Wilcoxon Rank) and specificity of 99.5% (adjusted *p* value from ${2\times 10}^{-16}$ into 0.0207 in Wilcoxon Rank) (Fig. 3B, Supplementary Material 2).

### Tool-specific performance to varying data complexity

Biomarker detection was evaluated using simulated HMP cross-sectional data with adjustments to assess challenges such as high dimensionality, unbalanced sequencing coverage, species abundance imbalance, outliers, and sparsity (Supplementary Material 3). With increasing the data dimensions, Lefse, edgeR, DESeq2, limma, metagenomeSeq, and ANCOM-BC2 maintained stable sensitivity (20–31%) and high specificity (86–99%) but showed limited false discovery rate (FDR) control, with uncorrected FDR reaching up to 84%. GEE-CLR-CTF, ANCOM, and ALDEx2 retained nearly perfect specificity with controlled FDR (< 5%) but showed declining sensitivity as dimensionality increased. With unbalanced coverage, most tools showed higher sensitivity with increasing the sequencing depth, except for ANCOM-BC2 which unexpectedly declined. ALDEx2 consistently showed high specificity (100%) with controlled FDR, while ANCOM exhibited fluctuating FDR levels. GEE-CLR-CTF was able to maintain FDR control (< 3%) and high specificity (99.8%).

Under the influence of outliers, most tools experienced sensitivity declines, yet only ALDEx2 and GEE-CLR-CTF managed to control FDR (< 5%) and maintain the high specificity. With increasing fold changes in sparsity, most tools’ sensitivity increased; however, only ALDEx2 and GEE-CLR-CTF consistently held FDR below 5%. With higher structural sparsity, the tools were able to achieve higher sensitivity yet mostly ALDEx2 and GEE-CLR-CTF were able to control the FDR levels. In terms of species abundance imbalance, ALDEx2 identified merely half of the low-abundance species identified by GEE-CLR-CTF, while the latter was able to capture both low and high abundances (6.25% and 12.5%, respectively).

### Development of the *metaGEENOME* R package

We integrated the GEE-CLR-CTF model inside R package called the *metaGEENOME*, aiming at simplifying the downstream analysis pipeline of microbial biomarkers. This package begins with preprocessing steps aligned with the GEE-CLR-CTF, eliminating low-prevalence, zero-inflated, and outlier data. Subsequently, it implements exploratory data analysis employing various visualization techniques including histograms for sequencing depth distributions, and taxa rank abundance curves. Alpha diversity assessments on rarefied datasets employ various methods, illustrated through taxa count boxplots. Significance testing for assigned variables utilizes non-parametric tests like the Wilcoxon rank-sum test for observed taxa and Shannon diversity. Beta diversity calculations use pre-assigned methods, represented via grouped variable boxplots and heatmaps.

*metaGEENOME* facilitates visualizing metadata variables using multiple ordination techniques, encompassing unconstrained and constrained methods (e.g., RCM—Reference Curve Multivariate), employing eigenvalues or distance-based methods like DCA, CCA, RDA, NMDS, MDS, and PCoA. Statistical identification of these variables involves hypothesis testing through distance matrices using PERMANOVA and adonis2, along with pairwise comparisons. The pipeline also incorporated the GEE-CLR-CTF approach for differential abundance (DA) analysis, with detailed supplementary figures. Finally, it compiles all analytical outputs into a comprehensive report for easy access and navigation.

## Discussion

The correct identification of differentially abundant bacterial species is a key step in biomarker discovery and remains a major challenge in 16S rRNA amplicon sequencing and shotgun metagenomics. This difficulty arises from the complex nature of microbiome data, which includes sparsity, imbalances between taxa, compositionality, high dimensionality, intra/inter-taxa correlation, missing data, and difficulties in modeling accurate data distributions [5–7]. Although several efforts have addressed these challenges through various normalization, transformation, and modeling techniques [12], most fail to achieve high statistical power and sensitivity without compromising control over the false discovery rates (FDR) and specificity [13, 42]. As a result, the accuracy of identifying differentially abundant species—and thus the integrity of detected microbial biomarkers—is often jeopardized. In this work, we introduce a novel statistical model coupling both normalization and transformation methods to resolve these challenges, through our introduced GEE-CLR-CTF approach.

A major obstacle in differential abundance (DA) analysis is the sparsity and inflation of zeros, often arising from outliers, structural zeros, or sampling bias. Our model effectively mitigates this by identifying and excluding outlier zeros while retaining biologically meaningful (sampling and structural) zeros for further analysis. This preserves data integrity without inflating false positives. Building on previously described methods [6], we evaluated count distributions within each group to distinguish typical data points from potential outliers. To address imbalances among taxa and variations in sequencing depth, we employed a normalization strategy using counts adjusted with trimmed mean of M-values (CTF). This method, robustly validated in both RNA-seq and microbiome datasets [19], minimizes the impact of between-sample biases while controlling for taxa imbalances. Additionally, the CLR (Centered Log Ratio) was applied to account for data compositionality [21], a known source of inflated false positives in microbiome studies [5]. To further enhance detection power, our model leverages the Generalized Estimating Equations (GEE) framework which is suited for longitudinal or clustered microbiome data as it accommodates correlations within repeated measures and varying distributions, offering reliable identification of differentially abundant taxa across cross-sectional and longitudinal data. [25, 27, 29, 30]. Lastly, we integrate taxa-variable interactions (local result) with a comprehensive global result, to better understand complex underlying relationships in the data.

We validate our GEE-CLR-CTF approach through comprehensive benchmarking based on two possible scenarios: cross-sectional and repeated-measures. This evaluation utilized both simulated data and real-life data, offering enhanced control over the ground truth in the former and capturing the complexity of real data in the latter. In the cross-sectional scenario, Lefse, MetagenomeSeq, and edgeR failed to adequately control FDR, with values ranging between 68.8 and 25.4%, and demonstrated low specificity (reaching a maximum of 94.3%), despite achieving higher sensitivity. This shortfall is likely due to the absence of restrictive transformation methods that alleviate compositional biases [43]. Conversely, tools incorporating transformation processes, such as ANCOM, ANCOM-BC2, and ALDEx2, exhibited better control FDR, ranging from 0.4 to 0.1%, as shown in the previous studies [13, 44], yet with specificity reaching 100% and sensitivity of 2.6%, 1.9%, and 4%, respectively. GEE-CLR-CTF achieved a high control of the FDR at 0.29%, alongside a modest enhancement in sensitivity at 6.9%, and specificity as high as 99.7%, demonstrating superior balance and consistent performance across both simulated and real datasets.

As for the repeated-measures assessment, several tools such as ALDEx2, limma-voom, and ANCOM-BC2 were not designed to handle the repeated-measures analysis properly and thus failed to achieve an acceptable level of sensitivity (not exceeding 1%). Other tools, namely ANCOM, MetagenomeSeq, DESeq2, and EdgeR, achieved inflated FDR levels ranging from 50 to 90%. GEE-CLR-CTF when compared to those tools was able to achieve the lowest FDR level (14.9%) while maintaining a high level of specificity (99.6%) and the highest sensitivity of 6.8%. These findings were observed to a lesser extent in our real data analysis which might be attributed to in challenging nature of ground truth identification in the real data. That being said, GEE-CLR-CTF was also capable of capturing the complexity of the microbiome data whilst having the lowest FDR. This demonstrated the capability of the GEE model to handle repeated measures analysis as well as the cross-sectional analysis, demonstrating its suitability in diverse scenarios, as previously reported in a different context [29].

DA tools often face a trade-off between sensitivity and FDR control. Our findings show that methods like MetagenomeSeq, edgeR, DESeq2, Lefse, and limma exhibit high sensitivity but lack strict FDR control. The GEE-CLR-CTF approach surpasses the other tools such as ANCOM and ALDEx2, which offer a better FDR control and account for the Poisson-like distribution of the data. To challenge the efficiency of these tools against various challenges within the microbial data, we conducted several modifications on our simulated data including high dimensionality, unbalanced sequencing coverage, species abundance imbalance, outliers, and sparsity biases**.** It was observed that Lefse, edgeR, DESeq2, limma, metagenomeSeq, and ANCOM-BC2 were not able to control the FDR (frequently exceeding 5%), despite showing high sensitivity (exceeding 10%) and specificity (reaching up to 90%). ANCOM struggled to control the FDR yet managed to achieve an average specificity of 99% with a lower average sensitivity of 6%. In contrast, both ALDEx2 and GEE-CLR-CTF maintained FDR control below 5%, with near-perfect specificity (100%). Notably, GEE-CLR-CTF achieved nearly twice the sensitivity of ALDEx2 across all simulation scenarios.

Despite these promising results, further refinement is needed, particularly in increasing statistical power while maintaining FDR control below 5%. As also emphasized by Mazer et al. [42], the issue of controlling the FDR is far more pressing for reliable microbial biomarkers identification. It is worth noting that at the time of writing, ANCOM-BC2 is still under active development; our benchmarking utilized version 2.4.0. Another issue that is yet to be addressed is the classification of the differentially abundant OTUs/ASVs, which poses a huge challenge considering the difficulty to delineate species boundaries with such a short region [45]. Yet the methods included in our study shed light on the broken promise of balance between sensitivity, specificity, and FDR levels in this field. Therefore, the GEE-CLR-CTF approach has been incorporated into a unified R package called metaGEENOME, covering an inclusive microbiome downstream analysis pipeline, which integrates crucial steps (from initial data filtering to exploratory analysis and hypothesis testing) providing a comprehensive platform for discovering microbial biomarkers.

## Conclusion

In conclusion, identifying differentially abundant bacterial species is critical for biomarker discovery but remains challenging due to the complexity of 16S rRNA and shotgun metagenomic data. While existing tools often trade off sensitivity for FDR control or vice versa, our evaluation confirms this imbalance—tools like MetagenomeSeq, edgeR, DESeq2, Lefse, and limma favor sensitivity but risk false positives, whereas ANCOM, ANCOM-BC2 and ALDEx2 control FDR at the cost of sensitivity. To address this, we developed GEE-CLR-CTF, a novel approach that combines established methods—GEE regression, CLR transformation, and CTF—with advanced zero handling. This integrated method demonstrated superior performance in both simulated and real datasets, achieving better FDR control, higher sensitivity, and strong specificity. Integrated into the metaGEENOME R package, GEE-CLR-CTF offers a robust, end-to-end platform for microbial biomarker discovery and hypothesis testing in both cross-sectional and longitudinal studies.

## Electronic supplementary material

Below is the link to the electronic supplementary material.

Supplementary Material 1: Comparison between the performance of ALR and CLR

Supplementary Material 2: Statistical validation results

Supplementary Material 3: Challenges evaluations

Supplementary Material 4: Cross-sectional and longitudinal parametric simulations. The diagram outlines data simulation and real evaluation. Simulated data mimics real abundance by introducing specific fold changes into distributions. It starts with data from the Human Microbiome Project and the Pelvic Irradiation Study. Real data is preprocessed by retaining Operational Taxonomic Units (OTUs) present in at least 5% of samples. Computational limits restrict the selection to 100 taxa, with new sub-data generated by shuffling samples. Parametric distributions like negative binomial and beta-binomial are applied. Taxa correlation is established using SpeiecEasi R package. Metadata groups are categorized into control and folded groups. Abundance values are derived by converting Pi numbers into theta values. True significant taxa nearby are determined by dividing data into subsets and comparing with the original dataset. Analysis begins with the American Gut Project (AGP) and ANTICIPATE study. Datasets are processed by trimming low-prevalence taxa, resulting in only 100 taxa in each subset. Each subset undergoes statistical tests, helping identify significant taxa, which are compared with the main dataset’s results to create the final confusion matrix

## Data Availability

The source code utilized for analysis can be publicly accessed on the GitHub repository located at: https://github.com/M-Mysara/metaGEENOME and The metaGEENOME package can be installed in R by remotes::install_github("M-Mysara/metaGEENOME"). Sequence data that support the findings of this study have been retrieved from the European Nucleotide Archive with the primary accession codes: PRJNA43021 (for HMP dataset; www.ncbi.nlm.nih.gov/bioproject/43021; [35]), PRJEB11419 (for AGP dataset; www.ebi.ac.uk/ena/browser/view/PRJEB11419; [36]), PRJNA685914 (for ANTICIPATE dataset; www.ncbi.nlm.nih.gov/bioproject/?term=PRJNA685914; [38]) and as for the Pelvic data it was retreived directly from the respective authors of the original work, [37].

## Abbreviations

OTU: Operational taxonomic unit
RLE: Relative log expression
TMM: Trimmed mean of M-values
CSS: Cumulative sum scaling
ALR: Additive log ratio
CTF: Counts adjusted with trimmed mean of M-values
CLR: Centered log ratio
GEE: Generalized estimating equations
TP: True positive (Correctly identifying a taxon as significant when it is indeed significant in the dataset)
TN: True negative (Correctly identifying a taxon as not significant when it is truly not significant in the dataset)
FP: False positive (Incorrectly labeling a taxon as significant when it is not actually significant in the dataset, type I error)
FN: False negative (Incorrectly failing to identify a taxon as significant when it is actually significant in the dataset, type II error)
FDR: False discovery rate
BH: Benjamini-Hochberg
DA: Differential abundance
