# Supplementary figures and images for: metaGEENOME: an integrated framework for differential abundance analysis of microbiome data in cross-sectional and longitudinal studies
Source: BMC Bioinformatics. 2025 Jul 21;26:189. doi: 10.1186/s12859-025-06217-x (PMC12281747; doi:10.1186/s12859-025-06217-x)

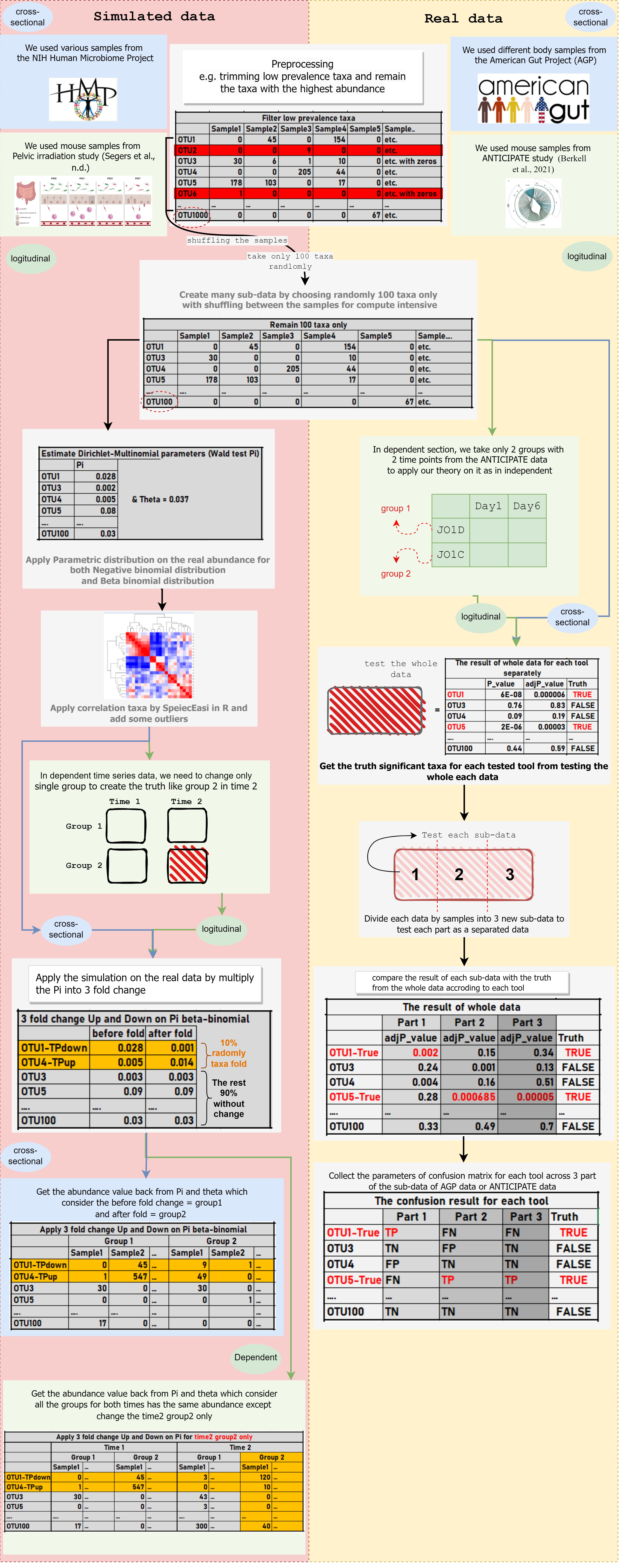

Supplement: Supplementary file 4 — Supplementary Material 4: Cross-sectional and longitudinal parametric simulations. The diagram outlines data simulation and real evaluation. Simulated data mimics real abundance by introducing specific fold changes into distributions. It starts with data from the Human Microbiome Project and the Pelvic Irradiation Study. Real data is preprocessed by retaining Operational Taxonomic Units (OTUs) present in at least 5% of samples. Computational limits restrict the selection to 100 taxa, with new sub-data generated by shuffling samples. Parametric distributions like negative binomial and beta-binomial are applied. Taxa correlation is established using SpeiecEasi R package. Metadata groups are categorized into control and folded groups. Abundance values are derived by converting Pi numbers into theta values. True significant taxa nearby are determined by dividing data into subsets and comparing with the original dataset. Analysis begins with the American Gut Project (AGP) and ANTICIPATE study. Datasets are processed by trimming low-prevalence taxa, resulting in only 100 taxa in each subset. Each subset undergoes statistical tests, helping identify significant taxa, which are compared with the main dataset’s results to create the final confusion matrix [file 12859_2025_6217_MOESM4_ESM.png]
